# CD4^+^FOXP3^+^ Regulatory T Cell Therapies in HLA Haploidentical Hematopoietic Transplantation

**DOI:** 10.3389/fimmu.2019.02901

**Published:** 2019-12-17

**Authors:** Antonella Mancusi, Sara Piccinelli, Andrea Velardi, Antonio Pierini

**Affiliations:** Hematology and Clinical Immunology and Bone Marrow Transplant Program, Department of Medicine, University of Perugia, Perugia, Italy

**Keywords:** regulatory T cells, allogeneic hematopoietic transplantation, tolerance, engraftment, graft-vs.-host-disease, graft-vs.-tumor effect

## Abstract

Since their discovery CD4^+^FOXP3^+^ regulatory T cells (Tregs) represented a promising tool to induce tolerance in allogeneic hematopoietic cell transplantation. Preclinical models proved that adoptive transfer of Tregs or the use of compounds that can favor their function *in vivo* are effective for prevention and treatment of graft-vs.-host disease (GvHD). Following these findings, Treg-based therapies have been employed in clinical trials. Adoptive immunotherapy with Tregs effectively prevents GvHD induced by alloreactive T cells in the setting of one HLA haplotype mismatched hematopoietic transplantation. The absence of post transplant pharmacologic immunosuppression unleashes T-cell mediated graft-vs.-tumor (GvT) effect, which results in an unprecedented, almost complete control of leukemia relapse in this setting. In the present review, we will report preclinical studies and clinical trials that demonstrate Treg ability to promote donor engraftment, protect from GvHD and improve GvT effect. We will also discuss new strategies to further enhance *in vivo* efficacy of Treg-based therapies.

## Introduction

CD4^+^FOXP3^+^ regulatory T cells (Tregs) are capable of suppressing the function of conventional CD4^+^ and CD8^+^ T cells (Tcons), B cells, NK cells and antigen presenting cells (APCs). They maintain tolerance to self and prevent autoimmune diseases, control excessive immune responses to allergens and pathogens, help maintain a balance with commensal microbial flora and the maternal tolerance to fetus. They have been shown to infiltrate tumors and suppress anti-tumor immunity (1–6). Recent studies have uncovered a role for bone marrow mouse Tregs in the maintenance of the hematopoietic stem cell (HSC) niche and in B cell lymphopoiesis (7, 8). Tregs develop in the thymus with a T cell receptor (TCR) repertoire that overlaps to some extent with that of Tcons (9–12). In addition, studies in mice reported that TGF-β and retinoic acid can induce differentiation of peripheral naïve CD4^+^ T cells into Tregs in response to antigenic stimulation (13–19). FOXP3 is a transcription factor of the forkhead winged helix family and is the lineage marker for both mouse and human Tregs (20–25), although human Tcons can express low levels of FOXP3 after activation (26, 27). FOXP3 deficiency causes lymphoproliferation and multi-organ autoimmunity in scurfy mice and a lethal X-linked syndrome with immune dysregulation, polyendocrinopathy, and entheropathy in humans (28–31). Recent studies in mouse models suggest that FOXP3 expression is not required to direct thymocyte development to the Treg cell lineage, but it is essential for Treg stability and function (32–34). TCR, IL-2, and TGF-β signaling can induce *Foxp3* transcription and are responsible for the maintenance and the function of thymic Tregs and the differentiation of Tregs in the periphery (35–38). Among others, FOXP3 represses the transcription of genes for IL-2 and other inflammatory cytokines, while it activates the transcription of *IL2RA, CTLA4*, and its own gene (39, 40). The α chain of the IL-2 receptor (IL-2Rα, CD25) and CTLA-4 are constitutively expressed by Tregs, while they are expressed on Tcons upon activation (1, 2, 41–44). Due to the intranuclear localization of FOXP3, CD25 is currently used to select Tregs for functional *in vitro* studies and cellular therapy, although it is not an exclusive marker. As Tregs do not produce IL-2, they depend on IL-2 produced by other immune cells, mainly Tcons (45). Tregs constitutively express the high-affinity receptor for IL-2 (IL-2Rα*βγ*_c_), thus they can efficiently compete for IL-2 with Tcons and NK cells (46–48). TCR stimulation activates Tregs that are capable of suppressing antigen-specific responses but can also exert bystander suppression (49). Tregs act through several mechanisms, including production of inhibitory cytokines such us IL-10 and TGF-β, cell-cell contacts, and cytolysis (5, 50). The inhibition of dendritic cell maturation and function is considered a core mechanism of Treg-mediated suppression. CTLA-4 on Tregs binds CD80 and CD86 on dendritic cells and inhibits maturation of APCs and co-stimulation of Tcons (51, 52).

Efforts have been made to exploit Treg function for the treatment of autoimmune and inflammatory diseases. Recently, clinical trials are evaluating Treg-based therapies in allogeneic hematopoietic transplantation (HCT) with promising results. Allogeneic HCT is a life-saving treatment for high-risk hematologic malignancies (53). After a conditioning regimen based on radiotherapy and/or chemotherapy, a bone marrow or a stem cell graft reconstitutes hematopoiesis and immunity of donor origin in the recipient. Residual host T cells that may have survived the conditioning regimen can recognize donor alloantigens and cause rejection. Similarly, donor alloreactive T cells recognize recipient alloantigens, eliminate malignant cells [graft-vs.-tumor (GvT) effect] and can prevent relapse. However, they also attack host normal tissues (mainly skin, gut and liver), causing graft-vs.-host disease (GvHD), that is a major cause of non-relapse mortality (NRM). Pharmacological immune suppression is widely used to prevent GvHD, but it also reduces the GvT effect. Separation of the GvT effect from GvHD is the main goal of the translational research in the field. In this setting, Tregs contribute to induction and maintenance of tolerance to alloantigens, facilitating engraftment and preventing the development of GvHD (54–56).

## Induction of Tolerance by Tregs in Preclinical Models of Allogeneic HCT

Mouse models of allogeneic HCT have provided evidence that Tregs suppress T cell alloreactions and can promote engraftment and help control GvHD. The role of Tregs in GvHD has been mostly investigated in MHC mismatched bone marrow transplantation. Pioneering studies demonstrated that CD4^+^CD25^+^ T-cell depletion from the bone marrow graft exacerbated GvHD. When additional CD4^+^CD25^+^ T cells, either freshly isolated or *ex-vivo* expanded, were infused, GvHD onset was delayed and even prevented to various degrees (57–60). One study showed that Tregs also protected from GvHD in a minor histocompatibility antigen-disparate, MHC matched setting (60). In a mouse model of fully mismatched T-cell depleted bone marrow transplantation, infusion of donor Tcons killed all the mice within 30 days of acute GvHD. When donor CD4^+^CD25^+^ Tregs were co-infused at a 1:1 ratio with Tcons more than 70% of mice were protected from lethal acute GvHD. Co-infusion of Tregs reduced the number of Tcons that could be recovered in lymph nodes and GvHD target tissues such as skin and gut, thus limiting Tcon expansion (61). Importantly, when mice were co-injected with a leukemia or lymphoma cell line, transfer of Tregs did not inhibit Tcon-mediated GvT effect (60–62). Moreover, Treg transfer preserved thymic and lymph node architecture and even accelerated donor lymphoid reconstitution to such an extent that mice survived lethal mouse Cytomegalovirus (CMV) infection (63). Treg homing to lymph nodes and target tissue is an important variable in GvHD prevention. In mouse models of GvHD, bioluminescence analyses showed that Tregs localized to peripheral lymph nodes and spleen in the first 24–48 h with a peak on day 4 after infusion, then they migrated to peripheral tissues. When Tregs were infused 2 days before Tcons, an unfavorable 1:10 ratio with Tcons still protected from GvHD at some extent (64). Moreover, when Tregs were eliminated *in vivo* 2 days after their transfer and even before Tcon injection, mice survived to GvHD (65). Finally, CD62L^−^ Tregs do not migrate to lymph nodes and are not capable of controlling GvHD (66, 67). Thus, Tregs migrate to lymph nodes and suppress Tcon proliferation early after transplant. However, Tregs could also suppress alloreactive Tcons in GvHD target tissues. One study showed that CCR5-deficient Tregs had reduced migration to mesenteric lymph nodes, liver, lung, and spleen and were less effective in preventing GvHD (68). In another study, CXCR3-transfected Tregs migrated better to liver, lung, and intestine and better controlled GvHD (69). Adoptively transferred third party Tregs also conferred protection from GVHD in mouse models of allogeneic transplantation, although they were less effective than donor Tregs. Third party Tregs survived for a shorter period, probably because they were rejected by donor Tcons. Thus, suppression of alloreactive T cells can also operate through MHC-independent mechanisms (65). Moreover, both radiation-resistant host Tregs and donor Tregs reduced the severity of chronic GvHD in a mouse model of bone marrow transplantation with a minor histocompatibility antigen mismatch (70). The capability of human donor Tregs to prevent and ameliorate GvHD caused by co-infused Tcons has been demonstrated in xenogeneic mouse models (71–73), and one of these studies also showed that the GvT effect was unaffected (73). Similar results were obtained with the infusion of third party human Tregs derived from umbilical cord blood and expanded before infusion (74). The same group also showed that fucosylation of expanded third party Tregs improved prevention of GvHD. In fact, the addition of a fucose formed the moiety found on P-selectin ligand on Treg cell surface, enhancing their persistence *in vivo* as a result of improved homing to the sites of inflammation (75).

Other studies focused more specifically on the role of Tregs in the engraftment of T-cell depleted bone marrow allografts. Joffre et al. demonstrated that when host Tregs were activated *in vitro* with donor allogeneic APCs, they inhibited host CD4^+^ and CD8^+^ T-cell mediated rejection of donor bone marrow graft, but not of a third-party bone marrow graft (76, 77). Another study showed that either donor or host CD62L^hi^, but not CD62L^lo^, *ex-vivo* activated Tregs inhibited rejection of MHC-mismatched bone marrow in sublethally irradiated mice (66). In a similar model, donor Tregs promoted engraftment without being activated *ex-vivo* (78). Steiner et al. showed that third-party Tregs, either naïve or *ex-vivo* expanded, enhanced engraftment of bone marrow allografts (79). Adoptive transfer of host-type Tregs can also induce mixed chimerism and tolerance to a fully mismatched bone marrow graft after a conditioning with short-course costimulation blockade (with CTLA-4 Ig and anti-CD40L antibody) and rapamycin, in the absence of cytoreductive treatment (80). Rapamycin is used because it limits activation and expansion of Tcons while promoting the expansion of Tregs (81). The ability of Tregs to promote engraftment is consistent with their role in the maintenance of the HSC niche, where they protect HSCs from autologous and allogeneic immune attack (7, 8). Tregs are also critical for tolerance induction to allogeneic HCT after reduced intensity conditioning regimens with total lymphoid irradiation and anti-thymocyte globulin (TLI/ATG). These regimens kill host-type Tcons while partially sparing Tregs, which increase donor HSC engraftment, cell cycling and differentiation (82, 83). These results support the use of TLI/ATG to establish mixed chimerism after allogeneic HCT in patients with hematologic malignancies (84).

## Treg-Based Therapies in One HLA Haplotype Mismatched Transplantation

In one HLA haplotype mismatched (haploidentical) hematopoietic transplantation, the high degree of HLA mismatch triggers strong host-vs.-graft and graft-vs.-host alloresponses. In the early 1990s, the combination of a myeloablative and immunosuppressive conditioning regimen with the infusion of a “mega-dose” of T-cell-depleted HSCs made haploidentical transplantation feasible and effective without the need for any post-transplant GvHD prophylaxis. The major limitation of this approach was delayed post-transplant immune recovery, which resulted in ~40% NRM, mainly due to infections (85–87). More recently, protocols of unmanipulated (T-cell replete) haploidentical transplantation have been developed. They are based on new strategies to control T-cell alloreactivity that also rely on Treg-induced tolerance (87, 88). A widespread and effective approach is the administration of high-dose cyclophosphamide following graft infusion (PTCy), which inhibits alloresponses while sparing donor HSCs (88–90). Donor Tregs are resistant to PTCy-induced cytotoxicity upon allogeneic HCT, because they express high levels of the enzyme aldehyde dehydrogenase, which is essential for *in vivo* detoxification of cyclophosphamide (91). Treg-mediated suppression plays an essential role in the prevention of GvHD in mouse models of allogeneic HCT with PTCy (91, 92). A recent study showed that PTCy induced functional impairment rather than elimination of alloreactive T cells and confirmed a role for Tregs in GvHD mouse models (93). Moreover, recovery of mature and functional natural killer (NK) cells is also impaired in patients undergoing haploidentical transplantation with PTCy (94, 95). Such effects could impair T cell- and NK cell-mediated leukemia killing after transplant. Importantly, clinical data suggest survival after haploidentical transplantation with PTCy is similar to that of patients undergoing HLA-matched sibling or unrelated HCT (96–102). Another trial used a rapamycin-based GvHD prophylaxis in order to promote *in vivo* expansion of Tregs and allow the infusion of unmanipulated haploidentical grafts (103).

In the setting of T-cell depleted haploidentical transplantation without post-transplant immunosuppression, the Perugia Bone Marrow Transplant Program is exploiting adoptive immunotherapy with freshly isolated donor Tcons and Tregs, in order to promote immune reconstitution while preventing GvHD (73, 87, 104, 105). CD4^+^CD25^+^ Tregs are isolated by a two-step immunomagnetic selection consisting of a negative CD19/CD8 selection, followed by a positive CD25 selection. The purity of CD4^+^FOXP3^+^ Tregs is around 70–80%. 2 × 10^6^ Tregs/kg are infused 4 days before 1 × 10^6^ Tcons/kg and about 10 × 10^6^ CD34^+^ cells/Kg. No pharmacological GvHD prophylaxis is given. The first study reported results in 28 patients with hematologic malignancies (24 in any complete remission and 4 in relapse at transplant). They received a conditioning regimen with TBI, thiotepa, fludarabine, and cyclophosphamide. Twenty-six patients engrafted. Treg/Tcon adoptive immunotherapy was associated with rapid reconstitution of B cells and of T cells with a wide repertoire. Compared with standard T-cell depleted haploidentical transplantation, reconstitution of pathogen-specific CD4^+^ and CD8^+^ T cells, and of mature NK cells was faster. The incidence of CMV reactivation was markedly reduced, with no CMV-related deaths. Two patients developed ≥grade 2 acute GvHD and no patient developed chronic GvHD. One patient (in chemoresistant relapse at transplant) relapsed. Thirteen patients died of NRM, 4 of them because of extra-hematological toxicity (104). The second study extended the analysis to 43 patients with high-risk leukemia in any remission, including 24 reported before. In order to reduce NRM, a lower dose of cyclophosphamide or anti-T cell antibodies in the place of cyclophosphamide were used in the conditioning regimen. Forty-one patients engrafted. NRM was 40% and fell to 21% in patients who had received anti-T cell antibodies as a part of the conditioning. Incidence of ≥grade 2 acute GvHD was 15% and only one patient developed chronic GvHD. Incidence of leukemia relapse was 5% and it was significantly reduced compared to the standard protocol of T-cell depleted haploidentical transplantation (73). Finally, in an updated analysis of a total of 60 patients with acute leukemia in any remission at transplant, incidence of acute grade II-IV GvHD and chronic GvHD were 15 and 3%, respectively. Five-year incidence of NRM was 35%. Five-year relapse incidence was as low as 12%, confirming preclinical data that Treg adoptive transfer does not impair T-cell immune reconstitution and the GvT effect (87). More recently, this strategy of adoptive immunotherapy with Treg/Tcon in haploidentical transplantation has been combined with a low-toxicity conditioning regimen based on total marrow and lymphoid irradiation (TMLI). TMLI provides high-intensity irradiation to bone marrow, spleen, and major lymph node chains, while sparing other tissues (106). This transplant protocol has been tested in acute myeloid leukemia patients who were aged or unfit to receive total body irradiation. Preliminary data indicate this combination provides promising results in terms of control of leukemia relapse and chronic GvHD/leukemia-free survival (107). Despite acute GvHD still occurs in a relevant fraction of patients, Treg/Tcon adoptive immunotherapy strikingly improves outcomes of T-cell depleted haploidentical transplantation thanks to a better chronic GvHD/leukemia-free survival.

## Treg-Based Therapies in HLA-Matched and Umbilical Cord Blood Transplantation

Since the number of freshly isolated Tregs to be used for cellular therapy is limited (5–10% of CD4^+^ T cells in peripheral blood), several protocols of expansion under good manufacturing practice have been developed and tested in clinical trials (108). Such expansion protocols preserve FOXP3^+^ Treg purity and suppressive function *in vitro*. Tregs can be isolated from peripheral blood or umbilical cord blood by immunomagnetic selection as described above, or by flow cytometric cell sorting. Tregs are usually incubated with anti-CD3/CD28 beads and high-dose IL-2 with or without rapamycin for around 2–3 weeks (108–112). Another option is the use of a modified K562 cell line, which expresses the Fc receptor CD64 to cross-link an anti-CD3 antibody, and CD86 to provide co-stimulation in the presence of IL-2 (113–115). Brunstein and colleagues used this approach to prevent GvHD after double-umbilical cord blood transplantation. Tregs were isolated by CD25 immunomagnetic selection from a third party cord blood unit that was 4–6/6 HLA matched with the patient, and expanded *in vitro* (114). In the last published clinical study, 3–100 × 10^6^ Tregs/kg were infused in 11 patients with various hematologic malignancies, who also received post-transplant immune suppression. Expanded Tregs were detectable in patients for a maximum of 14 days after the infusion. The incidence of grade II-IV acute GvHD at 100 days was 9% compared with 45% in contemporary controls with the same transplant protocol without Treg infusion. Chronic GvHD was 0% compared with 14% in controls. Incidence of infections, NRM and relapse, and disease-free survival were similar in patients infused with Tregs and in controls (114). Such studies suggest third party Tregs can be an alternative to donor-derived Tregs. However, donor and third party Tregs have never been compared in clinical studies for their efficacy in GvHD prevention. The infusion of donor expanded Tregs has been also tested for the treatment of acute and chronic GvHD in small series of patients after HLA-matched transplantation, with a clinical response in some patients (109, 111).

A recent phase I/II study investigated the infusion of fresh donor Tregs and Tcons in the setting HLA-matched transplantation in 12 patients with various hematological malignancies (116). Tregs were purified by CD25^+^ immunomagnetic selection, followed by flow cytometric cell sorting of CD4^+^CD127^lo^CD25^+^ cells (as Treg cells do not express the IL-7 receptor α subunit CD127 or express it at low intensity). Purity of CD4^+^FOXP3^+^ Tregs was over 90%. The first cohort of 5 patients received frozen Tregs but showed signs of GvHD, consistent with the reduced functionality of Tregs after cryopreservation and thawing (117). The other 7 patients received freshly isolated Tregs in combination with low-dose single-agent GvHD prophylaxis. No acute or chronic GvHD was observed (116).

## Treatment With Low-Dose IL-2 in Allogeneic HCT

A promising alternative to Treg adoptive transfer is low-dose IL-2 therapy, that has been shown to selectively activate and expand Tregs in several autoimmune and inflammatory settings, due to the constitutive expression of CD25 and factors related to IL-2 receptor signaling (118, 119). The group from Dana-Faber Cancer Institute and Harvard University investigated *in vivo* stimulation of Tregs with low-dose IL-2 for the treatment of steroid-refractory chronic GvHD after allogeneic HCT. A phase I study established that the maximum tolerated dose of IL-2 was 1 × 10^6^ IU/m^2^/day. During the 8 weeks of treatment, Treg counts and the Treg:Tcon ratio rose and 52% of patients had a clinical response (120). In a phase II study, 35 patients with steroid-refractory chronic GvHD were treated for 12 weeks. Two patients withdrew, 5 patients required dose reduction, and 61% of evaluable patients had a partial response to treatment. After a 4-week hiatus, low-dose IL-2 therapy could be extended for 2 years in patients with partial response or stable disease (121). The same group showed that low-dose IL-2 induced phosphorylation of signal transducer and activator of transcription 5 (STAT5) in Tregs but not in Tcons and preferentially expanded Tregs *in vitro*. Moreover, patients with severe chronic GvHD had constitutive phosphorylation of STAT5 in Tcons and high levels of IL-7 and IL-15. Treatment with low-dose IL-2 was associated with increased STAT5 phosphorylation in Tregs and decreased STAT5 phosphorylation in Tcons. This resulted in enhanced Treg proliferation, thymic export and resistance to apoptosis (122). Strategies to enhance efficacy of low-dose IL-2 therapy are under investigation and include combination with infusion of purified Tregs or extracorporeal photopheresis (123, 124). Another phase II study used ultra-low dose IL-2 for GvHD prophylaxis in 16 pediatric recipients of allogeneic HCT. Treatment started at a median of 28 days after transplant and consisted of 1–2 × 10^5^ IU/m^2^ three times per week for 6 or 12 weeks. This treatment was safe and it was associated with expansion of Tregs *in vivo*. None of the patients developed II-IV GvHD, while the incidence of acute GvHD was 12% in a control group of patients who did not receive IL-2 treatment (125).

## Perspectives

Recent clinical trials demonstrate that Treg-based therapies can effectively promote engraftment and prevent or treat GvHD after allogeneic HCT. However, these treatments fail to protect some patients from severe GvHD. Strategies to improve purity, enhance specificity, promote activation and control localization of Tregs are needed. Several experimental strategies are under investigation to achieve this goal.

Recent studies have focused on the generation of IL-2 compounds with enhanced selectivity for Tregs (126). One strategy is the use of anti-IL-2 antibodies in complex with IL-2 that allow for selective stimulation of T cell subsets. Boyman et al. showed that one anti-mouse IL-2 antibody prevented the binding of IL-2 to IL-2Rβγ_c_, but not to CD25 (IL-2Rα)_._ When complexed with IL-2, this antibody preferentially triggered the proliferation of Tregs (127). Subsequently, Trotta et al. reported the generation of a fully human anti-IL-2 antibody (F5111.2) that exerted the same effect on human Tregs when complexed with IL-2. Treatment with F5111.2-human IL-2 complexes was effective in preclinical models of autoimmune diseases and GvHD, and it did not affect immune response to mouse CMV (128). IL-2 can also be covalently linked to the antibody to generate a single-agent cytokine/antibody fusion that is more stable than the above immune complexes (129). Ward et al. designed a fusion protein of mouse IL-2 and CD25 that allows for a selective stimulation of Tregs *in vivo*. A treatment with this fusion protein delayed the development of diabetes in non-obese diabetic mice (130). An alternative strategy is engineering human IL-2 so that it preferentially binds the high affinity receptor IL-2Rα*βγ*_c_ and, consequently, preferentially activates Tregs (131, 132). Moreover, these IL-2 muteins are also designed to have a longer half-life and can provide a persistent stimulation of Tregs (130–132).

Another pleiotropic cytokine, TNF-α, has been recently shown to enhance Treg function. After the conditioning regimen, tissue macrophages release TNF-α, which activates donor alloreactive T cells that cause GvHD (133, 134). In fact, anti-TNF-α therapy with infliximab and etanercept is used to treat steroid-refractory chronic GvHD. However, some patients do not respond or even worsen (134–136). Tregs express higher levels of TNF receptor (TNFR) 2 compared with Tcons, and TNFR2^+^ Tregs are more suppressive than TNFR2^−^ Tregs (137–140). While TNFR1^−/−^ mice have defective immunity to infections and inflammatory response, TNFR2^−/−^ mice are affected by exacerbated inflammation (141). Several reports suggest that TNF-α/TNFR2 signaling is required for effective mouse Treg development in the thymus and optimal function *in vivo* (142–145). In mouse models of allogeneic bone marrow transplantation, GvHD control was abrogated when mice were infused with TNFR2 deficient Tregs or with a TNFR2 blocking monoclonal antibody (143). Moreover, *ex-vivo* priming of Tregs with TNF-α enhanced the effectiveness of GvHD prevention (144). Finally, expansion of radiation resistant host Tregs with a TNFR2 agonist reduced GvHD severity and improved survival (145). Importantly, the GvT effect was unaffected by Treg stimulation in these studies. *Ex-vivo* priming or expansion of Tregs with TNF-α or a TNFR2 agonist before adoptive transfer could improve prevention and treatment of GvHD. TNFR2 agonists could also be used to expand Tregs *in vivo* (146, 147). As Tregs express higher levels of TNFR2, they could be preferentially activated. However, Tcons upregulate TNFR2 expression upon activation and become more resistant to Treg-mediated suppression (148). Thus, the effects of *in vivo* TNFR2 signaling stimulation should be carefully evaluated. Another option is treatment with agonists of the costimulatory receptor TNFR superfamily 25 (Death receptor 3, DR3), which strongly stimulate Treg proliferation while weakly affect CD4^+^ T cell proliferation (149, 150). Treatment of donor mice with DR3 agonists reduced severity of GvHD in recipient mice of MHC-mismatched bone marrow transplantation, preserving GvT effects (151). Moreover, when recipient mice received a prophylactic treatment with a DR3 agonist, recipient-derived Treg expanded and severity of GvHD was reduced. In contrast, treatment of recipient mice after transplant favored donor Tcon alloreactions and worsened GvHD (152). In other studies, Treg expansion was induced in donors with the combination of a DR3 agonist and low-dose IL-2. The infusion of donor expanded Tregs ameliorated GvHD but did not affect GvT activity in both MHC matched and MHC mismatched bone marrow transplantation models (153–155). Recently, Copsel et al. combined this approach with the administration of inhibitors of bromodomain and extra-terminal proteins (BETi), which suppress expression of pro-inflammatory cytokines and other genes involved in T cell activation. They found that BETi EP11313 spared Tregs and that GvHD severity was reduced in mice treated with EP11313 and low numbers of donor Tregs expanded with DR3 agonist and low-dose IL-2 (156).

While current protocols of adoptive transfer use polyclonal Tregs, antigen-specific Tregs could be more effective without exerting a broad suppression of immune responses. Alloantigen-specific Tregs can be generated in the presence of allogeneic APCs or by TCR gene transfer. An alternative is the generation of chimeric antigen receptors Tregs (CAR-Tregs) (157). MacDonald et al. generated human HLA-A2-specific CAR-Tregs that prevented GvHD caused by HLA-A2^+^ Tcons in a xenogeneic mouse model. They were more effective than Tregs expressing an irrelevant CAR (158). A similar strategy was also used to prevent rejection after xenogeneic transplantation (159). Another study used Tregs expressing a CAR that binds Fluorescein isothiocyanate-conjugated monoclonal antibodies (mAbCAR-Tregs). Using tissue-specific antibodies, mAbCAR-Tregs could be directed to different sites, where they exerted their suppressive function (160).

Finally, several compounds are under investigation for the prevention and treatment of GvHD, such as Janus-activated kinase inhibitors and others. They share the ability to preferentially inhibit Tcon function while sparing Treg suppressive activity to various degrees (161). Thus, Tregs play a key role in many of the current and newly developed strategies to induce tolerance in allogeneic HCT.

## Conclusions

**Figure 1:**
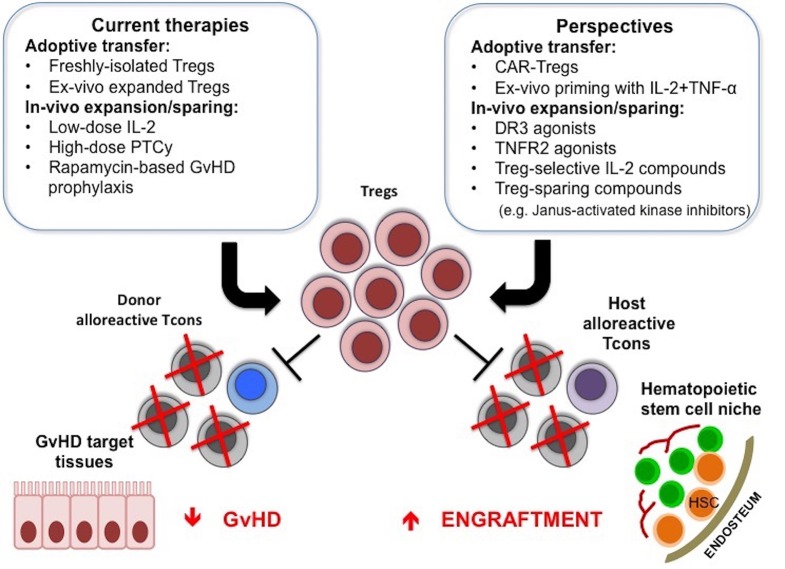
Treg-based therapies in allogeneic HCT. Adoptive transfer of Tregs or the use of compounds that can favor their function *in vivo* are used to promote donor engraftment and protect from GvHD after HCT. New strategies to further enhance *in vivo* efficacy of Treg-based therapies are under active investigation, and include CAR-Tregs, *ex-vivo* priming with IL-2 and TNF-α, TNFR2, or DR3 agonists, Treg-selective IL-2 compounds or compounds that inhibit Tcon function while sparing Treg suppressive activity.

Treg-based therapies have provided promising clinical responses in allogeneic HCT. These treatments have proven to be safe and are not associated with the side effects that could have been anticipated, such as increased susceptibility to infections and leukemia relapse. Efforts are ongoing to improve the effectiveness of these approaches, whether they are based on adoptive transfer of freshly isolated, expanded or modified Tregs or on the induction of Treg expansion and function *in vivo* (Figure 1).

To these days, treatment of patients with chemoresistant leukemia is still largely ineffective. In the present review, we discussed that Treg-adoptive transfer allows for a strong Tcon-mediated GvT effect while controlling GvHD. Although it requires further optimization, we believe this strategy is close to become an effective treatment for these patients.

## Author Contributions

AM and SP wrote the manuscript. AV reviewed the manuscript. AP wrote and reviewed the manuscript.

### Conflict of Interest

The authors declare that the research was conducted in the absence of any commercial or financial relationships that could be construed as a potential conflict of interest.
